# Characterising the Long-Term Language Impairments of Children Following Cerebellar Tumour Surgery by Extracting Psycholinguistic Properties from Spontaneous Language

**DOI:** 10.1007/s12311-023-01563-z

**Published:** 2023-05-15

**Authors:** Cheyenne Svaldi, Philippe Paquier, Stefanie Keulen, Henrieke van Elp, Coriene Catsman-Berrevoets, Annet Kingma, Roel Jonkers, Saskia Kohnen, Vânia de Aguiar

**Affiliations:** 1 Center for Language and Cognition, University of Groningen, PO box 716, 9700 AS Groningen, the Netherlands; 2 Clinical and Experimental Neurolinguistics (CLIEN), Brussels Centre for Language Studies (BCLS), Language, Brain and Cognition, Vrije Universiteit Brussel (VUB), Pleinlaan 2, 1050 Brussels, Belgium; 3 School of Psychological Sciences, Macquarie University, University Avenue, Macquarie Park, NSW 2109 Australia; 4 International Doctorate for Experimental Approaches to Language and Brain (IDEALAB), Newcastle University, Newcastle Upon Tyne, UK; Macquarie University, Sydney, Australia; University of Potsdam, Potsdam, Germany; University of Groningen, , Groningen, the Netherlands; 5 Centre for Research in Cognition and Neurosciences (CRCN), Université Libre de Bruxelles (ULB), Avenue Franklin D. Roosevelt 50, 1050 Brussels, Belgium; 6 Department of Translational Neurosciences (TNW), Universiteit Antwerpen (UA), Universiteitsplein 1, 2610 Antwerp, Belgium; 7 Department of Paediatric Neurology Erasmus Medical Centre, Sophia Children’s Hospital Rotterdam, Dr Molewaterplein 40, 3015 GD Rotterdam, the Netherlands; 8 Department of Paediatrics, University Medical Centre Groningen, Hanzeplein 1, 9700 RB Groningen, the Netherlands

**Keywords:** Cerebellum, Postoperative cerebellar mutism syndrome, Spontaneous language, Posterior fossa surgery, Word properties

## Abstract

Following cerebellar tumour surgery, children may suffer impairments of spontaneous language. Yet, the language processing deficits underlying these impairments are poorly understood. This study is the first to try to identify these deficits for four levels of language processing in cerebellar tumour survivors. The spontaneous language of twelve patients who underwent cerebellar tumour surgery (age range 3–24 years) was compared against his or her controls using individual case statistics. A distinction was made between patients who experienced postoperative cerebellar mutism syndrome (pCMS) and those who did not. Time since surgery ranged between 11 months and 12;3 years. In order to identify the impaired language processing levels at each processing level (i.e., lexical, semantic, phonological and/or morphosyntactic) nouns and verbs produced in the spontaneous language samples were rated for psycholinguistic variables (e.g., concreteness). Standard spontaneous language measures (e.g., type-token ratio) were calculated as well. First, inter-individual heterogeneity was observed in the spontaneous language outcomes in both groups. Nine out of twelve patients showed language processing deficits three of whom were diagnosed with pCMS. Results implied impairments across all levels of language processing. In the pCMS-group, the impairments observed were predominantly morphosyntactic and semantic, but the variability in nature of the spontaneous language impairments was larger in the non-pCMS-group. Patients treated with cerebellar tumour surgery may show long-term spontaneous language impairments irrespective of a previous pCMS diagnosis. Individualised and comprehensive postoperative language assessments seem necessary, given the inter-individual heterogeneity in the language outcomes.

**Supplementary information:**

The online version contains supplementary material available at 10.1007/s12311-023-01563-z.

## Introduction

Cerebellar tumours are the most common paediatric brain tumours [1, 2]. The five-year survival rate of patients treated for a cerebellar tumour steadily increases [2, 3], probably due to treatment improvements [4, 5]. This increased survival rate draws attention to the wide range of acute and long-term sequelae observed in survivors, which are partly attributable to the tumours, and partly to the necessary surgical and post-surgical treatments provided. Understanding the exact nature of possible detrimental late effects of such treatments is essential in providing long-term support for this group, including language rehabilitation [6].

Following cerebellar tumour resection, impairments have been observed across all language processing levels (i.e., semantic, lexical, phonological, morphosyntactic, pragmatic), including word-finding difficulties [7–9], pragmatic deficits [10] and agrammatism [8, 11, 12]. When these language impairments are preceded by mutism (i.e., a temporary absence of speech commonly accompanied by severe emotional lability and neurological deficits), this cluster of symptoms is referred to as postoperative cerebellar mutism syndrome (pCMS) [13]. This syndrome occurs in approximately 25 to 35% of children who have cerebellar tumour surgery [14, 15]. Postoperative language impairments may also occur without being preceded by pCMS and the comparison between children who did and did not suffer from pCMS has yielded inconsistent results in the patterns of impairment and their severity [7, 16, 17]. To the best of our knowledge, no study has tried to identify impairments across all language processing levels. Results of this type of assessment would help guide language rehabilitation on an individual level and could shed further light into the nature of the language impairments in children who had cerebellar tumour surgery. It should be noted, however, that this population is already burdened with many other medical/psychological assessments after neurosurgery, highlighting the need for an ecologically valid assessment that gives an overview of the performance on multiple language areas while reducing testing time. To this end, the present study evaluates language impairments using spontaneous language.

Most of the data that currently exist on postoperative language outcomes in cerebellar tumour patients come from formal tests. Yet, spontaneous language analysis has been reported to be more ecologically valid than formal tests [18]. In addition, by examining multiple levels of language processing simultaneously, spontaneous language tasks can be used as a quick assessment when the patient is too tired for a comprehensive formal assessment. The assessment of spontaneous language is considered relevant in linguistic studies to differentiate between children with a developmental language disorder and typically developing peers [19, 20] and can classify different types of post-stroke aphasia [21, 22].

Reports on the spontaneous language outcomes in children treated with cerebellar tumour surgery are scarce, but have aided in documenting morphosyntactic (e.g., the omission of grammatical elements) [11, 12, 16], lexical-semantic (e.g., a reduced lexical diversity) [16] and/or pragmatic impairments [22]. The benefits for our understanding of language difficulties in cerebellar tumour survivors notwithstanding, studies that addressed spontaneous language generally looked at quantitative measures, such as lexical diversity (e.g., type-token ratio) which reflects vocabulary size [10, 11] and syntactic complexity [11, 16, 23]. However, these standard measures are too coarse to provide a deeper understanding of the nature of the language deficits in children after treatment for cerebellar tumours. Given that such measures have provided inconsistent results in previous research with children with cerebellar tumours [10, 11, 16], it is unclear if these standard measures alone are successful in differentiating patients with language problems from their controls. Also, none of these spontaneous language measures or previously used formal tests in this clinical population assess more than three aspects of language processing.

In this work, we introduce a novel and comprehensive approach to detect spontaneous language deficits. We conduct a detailed examination of psycholinguistic properties reflecting four linguistic levels, namely, phonological, lexical, semantic and morphosyntactic processing alongside standard spontaneous language measures (e.g., type-token ratio). We include a wide range of spontaneous language variables to determine which variables may be successful in differentiating patients from their controls. Also, we use these variables to characterise the nature of the deficits in our patient group. The rationale for adopting this method to cerebellar tumour survivors is based on previous work with similar populations.

### Word Properties: a Window Into the Processing Nature of Language Impairments

According to Shallice’s (1988) critical variable approach, word properties or psycholinguistic variables (e.g., imageability, word frequency) can influence both language comprehension [24–26] and production [27–29]. Furthermore, certain word properties can influence processing at specific linguistic levels [24, 28, 30]. When adopting the critical variable approach to spontaneous language analysis, the psycholinguistic variables extracted from the produced words may unravel the nature of observed language impairments [31, 32]. In this study, nine word properties that are extensively described in adults with post-stroke aphasia [25, 28, 31, 33] and children with a developmental language disorder [32, 34, 35] were extracted from the produced nouns and verbs in spontaneous language. This approach has already been used in studies with verbal fluency, for example in adults with primary progressive aphasia [30], and post-stroke aphasia [36] and in one spontaneous language study in bilingual children with developmental language disorder [32]. In what follows, the included psycholinguistic variables are outlined per level of language processing. These were selected to rate every patient’s ability to produce spontaneous language.

#### Semantic Variables

We selected three variables to assess participants’ ability to produce semantic properties of spoken language: imageability, concreteness, and verb instrumentality. Imageability represents the degree to which a concept gives rise to a mental image or sensory experience [30]. For example, “cat” has a high imageability rating, while “thought” has a low imageability rating. Concreteness, on the other hand, reflects the degree to which a concept is perceptible (e.g., “car” is more concrete than “happiness”) [30]. Verb instrumentality indicates if an action requires an instrument (not a body part). For example, the verb “to cut” requires scissors for the action to be completed, while the verb “to throw” does not require a tool or instrument. Effects of these word properties may reflect impairments of semantic processing (i.e., the activation of meanings in response to concepts or ideas), for example reduced stored knowledge within semantic representations [28, 30, 31]. Words of a higher imageability or concreteness have been reported to be easier to process than words of low imageability or concreteness in both people with post-stroke aphasia [28, 31] and in some children with a developmental language disorder [37]. Verb instrumentality has been reported to have both a facilitating and inhibitory effect on verb retrieval in people with post-stroke aphasia [33, 38]. In children with a developmental language disorder, non-instrumental verbs have been shown to be easier to name than instrumental verbs [35]. In spontaneous language, individuals with a semantic processing impairment may thus produce more imageable and/or concrete and more instrumental or non-instrumental verbs compared to their controls.

#### Lexical Variables

Three psycholinguistic variables were selected to investigate the lexical properties of spontaneous language, more specifically word frequency, age of acquisition (AoA) and phonological neighbourhood. Word frequency is obtained by counting the number of times a word appears in a corpus^1^ (e.g., “home” occurs many times in a corpus while “forklift” occurs much less) [39]. AoA is the age at which a word is learned in the written or spoken form. For example, “cookie” is learned at a young age, while “globe” is acquired later [40]. Word frequency and AoA have been reported to be negatively correlated (e.g., “to eat” has a low AoA but a high frequency). Phonological neighbourhood, finally, refers to the number of phonologically similar words created by substituting one phoneme of a target word [41]. For example, “book” has a high number of phonological neighbours (e.g., “look”, “bock”, “bush”) while “helicopter” has no phonological neighbours [42]. These three psycholinguistic variables have been consistently reported and can be indicative of deficiencies of the phonological output lexicon (i.e., the storage of spoken word forms) or lexical retrieval from the semantic system [24, 25, 28]. It has been proposed that high-frequency words with a lower AoA are easier to retrieve from the phonological lexicon and evoke fewer errors [27, 28]. However, AoA has also been argued to reflect language processing at the semantic level, considering that later acquired words have less dense semantic representations and are thus more vulnerable to impairment [24, 31]. A higher number of phonological neighbours has been reported to be a possible marker of developmental language disorders [32] and the number of phonological neighbours can hinder spoken word production [41].

#### Phonological Variables

One psycholinguistic variable was chosen to assess participants’ ability to process phonological properties of spoken language. Word length in phonemes (e.g., “cook” has three phonemes /*c-oo-k/*) was selected since this is the only property that could be extracted from the spontaneous language samples and of which effects were consistently reported in the literature. Word length effects can indicate deficiencies of phonological encoding. This is the assembly of phoneme strings before articulation [43]. For example, children with a developmental language disorder have been reported to have more difficulties with repeating non-words of an increasing length than typically developing children [34]. The predominance of shorter words can also reflect deficits in phonological short-term memory (i.e., the temporary storage of speech sounds).

#### Morphological and Syntactic Variables

Finally, two psycholinguistic variables were selected to investigate the syntactic (i.e., verb transitivity) and morphological (i.e., verb regularity) properties of spontaneous language. Transitive verbs are verbs that require a direct object. For example, in the sentence “the girl carries the bag” the verb “to carry” takes the direct object “the bag”. In contrast, intransitive verbs do not require an object. For example, in the sentence “the girl swims” the verb “to swim” does not take a direct object [38, 44]. Regularity is determined based on the patterns of inflection of a verb, related to, for example, tense and aspect. In English, for example, regular verbs retain the verb stem with the addition of the suffixes *-d* or *-ed* in the past tense (e.g., “jump-jumped”). Irregular verbs, on the other hand, do not follow this typical inflection as the vowel of the verb stem changes in the past tense (e.g., “drink-drank”). We selected these variables because they reflect morphological and syntactic processing on a word level. Effects of these variables have been reported to be indicative of a morphosyntactic impairment [38, 44–47]. For example, in adults with post-stroke aphasia and children with a developmental language disorder, transitive verbs have been reported to be more difficult to produce than intransitive verbs [38, 44, 45], but the opposite pattern has been found as well [46]. Although few studies have addressed the influence of verb regularity on language production, irregular verbs have been reported to be more prone to errors in children with a developmental language disorder [47] and children have been shown to be sensitive to verb regularity during language acquisition [48, 49]. Further, the cerebellar involvement in noun regularity has recently been described [50].

These psycholinguistic variables can help us thus understand the nature of the spontaneous language impairments in children with cerebellar tumours. For example, while previous studies reported lexical and morphosyntactic difficulties in children with posterior fossa tumours, semantic processing was never investigated in children after cerebellar tumour surgery [51, 52]. It was, however, associated with cerebellar functioning in individuals without neural damage and thus research is also warranted in case of cerebellar lesion. Also, psycholinguistic variables could provide us more insights into the differences in the nature of the spontaneous language impairments in children with and without pCMS. The few studies that compare postoperative spontaneous language outcomes in children with and without pCMS, generally report language impairment in both groups [11, 53]. Further, a preoperative reduction of the mean length of utterance (MLU; a measure of syntactic complexity) has been indicated as a possible prognostic factor for the development of pCMS, with morphosyntactic impairments persisting in the postoperative phase [11, 23, 54].

In summary, spontaneous language analysis is a powerful way to analyse language skills. Yet, to the best of our knowledge, spontaneous language has been underutilised in the study of postoperative language impairments after cerebellar tumour resection. The aim of the present study is twofold (1) to determine which spontaneous language variables can differentiate between ‘patients after cerebellar tumour surgery’ and ‘control speakers’ and (2) to characterise the nature of the deficits in our patient group across four linguistic levels. For the first aim, we conducted a principal component analysis (PCA) to select the relevant variables in our data and then conducted an individual patient analysis, comparing scores on these variables between patients and controls. For the second aim, we also differentiated between patients who experienced pCMS and patients who did not. This is the first study to evaluate four linguistic levels and is the most comprehensive study of spontaneous language in this clinical population.

## Methods

### Participants

#### Patient group

The data for this study were collected as part of a larger project that also investigated cerebellar-induced motor speech impairments [53, 55]. The patient group was recruited initially through a review of the neuropsychological and neurolinguistic records of children who underwent cerebellar tumour surgery at the Erasmus Medical Centre/Sophia Children’s hospital in Rotterdam between 1995 and 2007. The inclusion criteria were (1) surgical resection of a cerebellar tumour before the age of eighteen; (2) no reported history of preoperative developmental, neuropsychiatric, learning or language deficits; (3) no history of neurological or motor impairments before tumour diagnosis; (4) the presurgical presence of a cerebellar tumour as confirmed by Magnetic Resonance Imaging (MRI) and (5) availability of premorbid developmental data and a recorded speech sample of at least three hundred words which is the minimum for a reliable analysis [19, 22, 56]. Patients who experienced pCMS and patients who did not were included in this study. MRI scans were conducted pre- and postoperatively (within three months of assessment) to determine tumour characteristics (i.e., location, type, and size), the surgical incision site and the degree of tumour removal. The presence of pre- or postoperative hydrocephalus was determined with the bicaudate index (BI) for ventricle dilatation [57]. We distinguished between no (BI < 0.19), mild (BI = 0.19–0.26) and severe hydrocephalus (BI > 0.26) [58].

Thirty-two children and young adults treated with cerebellar tumour surgery were identified. Of this group, fourteen patients were excluded because their files were incomplete. More specifically, language data were absent for thirteen patients and medical information was incomplete for one patient. One other patient was excluded because of a premorbid developmental delay and one because no surgical resection was done. Four patients were excluded because the recorded speech sample contained less than three hundred words. The mean age at assessment of the final group of twelve participants was 11;3 years (*SD* = 6;3 years; range = 3–24;2 years) and consisted of five females and seven males. Mean age at surgery was 7;2 years (*SD* = 4;7 years; range = 2;1–17;9 years), meaning that patients were on average at 4;8 years (*SD* = 3;8 years; range = 0;11–12;3 years) post-surgery. Most patients were operated for a medulloblastoma (5/12) or pilocytic astrocytoma (5/12). Five patients suffered from pCMS. The individual demographic, tumour and tumour treatment characteristics of the patient group are provided in Table 1.

**Table 1:** Demographic, tumour, and tumour treatment characteristics of the participants who underwent cerebellar tumour surgery (n = 12)

Group	Case	Gender	Age at assessment (yy;mm)	Time since surgery (yy;mm)	Duration mutism (days)	Tumour type	Tumour diameter (cm)	Tumour location	Hydrocephalus (BI)		Treatment	Extent of resection	Age controls (*M* (*SD*)) (yy;mm)
									Pre-op	Post-op			
*pCMS*	*P6*	M	6;0	2;10	29	PA	3.5	4^th^ V + pons + RCH	0.26*	0.10	S + C	Subtotal	6;4 (0;3)
	*P8*	F	7;8	2;1	51	PA	5	Vermis + 4^th^ V	0.27*	0.03	S	Total	7;10 (0;4)
	*P2*	F	8;10	4;2	21	MB	4.8	Vermis	0.24*	0.19	S + C + R	Subtotal	7;10 (0;4)
	*P7*	M	19;9	2	70	MB	4.3	Vermis	0.25*	0.11	S + C + R	Total	21;6 (1;11)
	*P16*	M	24;2	12;3	152	MB	4.1	Vermis	0.19	0.14	S + C + R	Total	21;6 (1;11)
*no pCMS*	*P17*	M	3;0	0;11	N.A	EP	3.5	Vermis + LCH	0.20*	0.10	S + R	Subtotal	3;4 (0;3)
	*P25*	M	6;7	4;2	N.A	MB	5	Vermis	0.15	0.14	S + C + R	Subtotal	6;4 (0;3)
	*P24*	M	8;1	1;2	N.A	MB	4.6	Vermis	0.28*	0.15	S + C + R	Subtotal	9 (0;4)
	*P20*	M	10;2	2;4	N.A	EP	2.3	Vermis	0.10	0.14	S + R	Total	9;11 (0;4)
	*P23*	F	11;1	1;10	N.A	PA	6.3	Vermis + LCH	0.28*	0.13	S	Total	11;10 (0;4)
	*P26*	F	11;5	7;4	N.A	PA	4.2	Vermis	0.43*	0.05	S	Total	11;10 (0;4)
	*P22*	F	18;3	8	N.A	PA	4	Vermis + 4^th^ V + LCH	0.37*	0.1	S	Subtotal	18;5 (0;5)

*pCMS* = postoperative cerebellar mutism syndrome; *P* = patient; *yy;mm* = years;months; *N.A.* = not applicable; *PA* = pilocytic astrocytoma; *MB* = medulloblastoma; *EP* = ependymoma; *4*^*th*^* V* = fourth ventricle; *RCH* = right cerebellar hemisphere; *LCH* = left cerebellar hemisphere; *BI* = bicaudate index; * = hydrocephalus; *S* = surgery; *C* = chemotherapy; *R* = radiotherapy

#### Control group

The control group consisted of thirty-nine individuals with no history of language impairment (24 males, 15 females) who were matched for age and gender with each patient. Mean age of the control group was 11;1 years (*SD* = 5;11 years; range = 3;0 – 24;3 years) at the time of assessment. The controls were recruited via primary schools and a speech and language therapy practice in the north of the Netherlands. The latter included siblings of children treated at the practice but without any speech and language impairments. The recruitment target was five control participants per patient with a cerebellar tumour, to allow statistical comparisons between an individual’s result and a matched control group [59]. Inclusion criteria were: (1) no history of neurological impairments and; (2) no history of developmental, neuropsychiatric, learning and/or language deficits. All participants who were twelve years of age or older gave written informed consent. Also, all parents of participants less than eighteen years of age gave consent. Ethical approval for the collection of control data was obtained from the Research Ethics Committee (CETO) from the faculty of arts of the University of Groningen (review number 76303408).

To ensure that none of the controls had a language impairment or delay, language was assessed using formal tests according to age in addition to the spontaneous language assessment. For children of five years or younger, the Core Language Score of the Dutch version of the Clinical Evaluation of Language Fundamentals Preschool (CELF-Preschool-2-NL) [60] was determined. For children between six and sixteen years of age, the Core Language Score of the Dutch version of the CELF (CELF-4-NL) [61] was calculated if a child was recruited via the speech therapy practice. A standard score between 85 and 115 was deemed within the normal range. If a child was enrolled in the study through contact with a primary school, the scores of the language subtests of the Cito tests were examined. These are tests routinely administered to children in the Netherlands to predict future learning success [62]. Children receive a score of A to E (A = highest 25% of achieving pupils; B = 25% at to above the national average; C = 25% at the national average to well below; D = 15% well below to far below national average; E = 10% lowest performing pupils) based on standardised assessments of reading comprehension, reading fluency, spelling and mathematics. Children with a score ranging from A to D on the subtests ‘technical reading’, ‘spelling’, ‘vocabulary’ and ‘reading comprehension’ were included. Parents of children between the ages of six and sixteen years of age also completed the Children’s Communication Checklist (CCC-2-NL) to exclude the presence of language impairments [63]. To ensure that a control had no history of any other deficits, the participant and/or his/her parents filled in a questionnaire considering their demographic information, language development and history of language and neurodevelopmental impairments. This questionnaire was designed by the authors for the purpose of this study.

### Procedure

The linguistic data were collected during systematic clinical neuropsychological assessments which are offered to children who have been treated for a central nervous system tumour. According to the Dutch Child Oncology Group follow-up protocol (SKION), these assessments take place during a follow-up period of one to three years, and after that, every three years after tumour resection. The speech samples were either recorded or videotaped for future intra-subject comparison.

Spontaneous language was collected through an open-ended conversation about daily activities (i.e., school, family, hobbies, pets) and by describing three pictures [64–66]. Spontaneous language was video recorded or audiotaped and transcribed by four independent researchers following a detailed transcription protocol. This protocol was largely based on the Spontaneous Language Analysis Procedure (STAP) [67] and Analysis of Spontaneous Language in Aphasia (ASTA) [56]. Some changes were made, however, to fit the clinical group and goals of the analysis. Thirty percent of the samples were transcribed by the first and fourth authors (both native speakers of Dutch) to calculate inter-rater reliability.

Participants had to produce at least three hundred words (all words starting from the beginning of the sample, including repetitions, content words and function words) to be included in the study. This sample length has been reported to provide reliable measures in samples of children with a developmental language disorder and adults with post-stroke aphasia [19, 22]. If the beginning of the speech sample was not representative of the communicative abilities of the participant (e.g., the participant was very shy at the beginning of testing), the word count started after the first fifty words were produced. Part of the data of the children who underwent cerebellar tumour surgery were gathered in 2007. An auditory-perceptual speech analysis of the sample was published previously [55]. Additional control data were collected in 2020–2021. The procedure was different for controls, for whom language assessment consisted of two sessions instead of one.

### Data coding

Data coding was done separately for the conversation and picture description data since these are two different methods of spontaneous language elicitation. This distinction resulted in shorter samples (10–50 utterances) than the three hundred word minimum, but this has been reported to be sufficient to obtain reliable results for the included measures [68, 69]. The length of the samples of the conversational data was based on the total number of utterances produced by every patient (excluding minimal responses), to which the samples of the controls were matched. For the picture analysis, the complete descriptions of the three pictures were analysed.

A total of twenty-one standard (i.e., lexical diversity, syntactic complexity and accuracy) and psycholinguistic spontaneous language variables were included. In Table 2, an overview of the variables per level of language processing is provided. We included five semantic, nine lexical, two phonological and five morphosyntactic variables.

**Table 2:** Overview of the included spontaneous language variables per level of language processing

Level of language processing	Standard spontaneous language measures	Psycholinguistic variables
*Semantic*	-	Concreteness*
		Imageability*
		Verb instrumentality (proportion)
*Lexical*	Lexical diversity (TTR)*	Age of acquisition*
	Lexical accuracy (percentage)	Word frequency*
		Phonological neighbourhood size*
*(Lexico-)phonological*	-	Word length (in phonemes)*
*Morphosyntactic*	Mean utterance length (in words)	Verb transitivity (syntactic, proportion)
	Grammatical accuracy (percentage)	Verb regularity (morphological, proportion)
	Finiteness index (percentage)	

* = Separate variable for nouns and verbs; *TTR* = Type-token ratio

Five standard language measures were included, reflecting lexical and morphosyntactic processing. Lexical diversity was measured by type-token ratio, which was calculated by dividing the total number of produced nouns/verbs by the number of tokens (i.e., the number of different nouns/verbs). Syntactic complexity was measured using MLU or the average number of produced words per utterance. To evaluate lexical/grammatical accuracy or the correctness of the produced utterances, the proportion of lexically (e.g., no neologisms, word choice errors) and grammatically (e.g., no errors on sentence structure, verb conjugation) correct utterances was calculated. Finally, finiteness index (i.e., the proportion of correctly inflected finite verbs) evaluated finite-verb-conjugation.

All nouns and verbs were extracted from the spontaneous language samples to obtain the values for the psycholinguistic variables. Ratings for imageability, concreteness and AoA were based on self-ratings by speakers, while phonological neighbourhood and word frequency were objectively quantified. Values for imageability were acquired from the database of Van Loon-Vervoorn [70]. Values for concreteness and AoA were obtained from the databases by Brysbaert et al. [40]. The number of phonological neighbours and the word length of each noun or verb was retrieved from the Dutch interface of the Cross-Linguistic Easy-Access Resource for Phonological and Orthographic Neighbourhood Densities (DutchPOND) [42]. Finally, lexeme frequency ratings were obtained from the SUBTLEX-NL database [39] that is also embedded in DutchPOND. More specifically, the SUBTLEXWF value was extracted for every noun or verb. This value represents the frequency of occurrence of a word per million words and is independent of corpus size [39]. Verbs were coded for instrumentality, transitivity and regularity based on the linguists’ experience (first to third and seventh to ninth authors) and previous literature [33]. For every participant, the mean and standard deviation for the ratings of every psycholinguistic variable were calculated (see Supplementary materials). Data coding was performed by two experienced researchers and 30% of samples were coded by both researchers. Inter-rater reliability was calculated for the data coding of these samples using Cohen’s Kappa. Disagreement in data coding was resolved by discussion between raters.

### Statistical analyses

Statistical analyses encompassed a PCA and individual comparisons using Crawford’s modified t-tests [59]. A separate analysis was conducted for the conversation and picture data. First, a PCA^2^ was carried out for each level of language processing to reduce the number of variables into overlapping components. Second, comparisons between each patient’s data and the average of his or her corresponding matched control group were performed separately for each of the components identified with PCA. In what follows, the statistical analyses are explained in further detail.

The suitability of the data for the PCA was examined with the Kaiser–Meyer–Olkin Measure of Sampling Adequacy (KMO) and Barlett’s test of sphericity. For the data to be suitable, a KMO statistic of at least 0.50 was required for every variable along with a significant Bartlett’s test [71]. Variables scoring below the 0.50 KMO criterion were removed from the PCA. An orthogonal Varimax rotation was employed to increase the interpretability of the data. The number of principal components for every subset of variables was determined based on their eigenvalues (i.e., > 1.0), the proportion of explained data variance (i.e., at least 70%) and the interpretability of the components (e.g., variable loadings > 0.45; previous literature e.g. [24, 28],).

Heterogeneity was expected in the language outcomes of the patient group [72]. Therefore, each score of every patient was compared to scores of five age- and gender-matched control speakers. Modified t-tests were already employed in this clinical population [73]. All analyses were performed in Rstudio v1.4.

## Results

Below, we will detail the PCA (aim 1) and then the individual comparisons per patient (aim 1 and 2). The language evaluations are described separately for the five patients who suffered from pCMS and the seven patients who did not receive this diagnosis. P6 was excluded from the conversation data and P2 from the picture description data. These patients produced less than five utterances on the respective spontaneous language tasks, rendering their outcomes unreliable for that task. Cohen’s Kappa indicated a moderate to substantial agreement (range = 0.58–0.73) between researchers based on 30% of the spontaneous language samples that were coded by two researchers. The main sources of disagreement were determining if a sentence was lexically correct (0.58) and the calculation of the finiteness index (0.59).

### Principal Component Analysis

A PCA was performed for every subset of variables (i.e., semantic, lexical and morphosyntactic) to make the analysis more manageable in relation to the number of variables while maintaining most (> 70%) of the data variance. Since only two phonological variables were included in the analysis, this subset of variables was not suitable for the PCA. Even though the literature is divided on this issue, AoA was included as a lexical variable for the PCA [24, 28]. Several variables were excluded to fulfil the KMO criterion. For the conversation data, the lexical variables ‘phonological neighbourhood nouns’ and ‘lexical accuracy’ and the morphosyntactic variable ‘finiteness index’ were removed. For the picture data, the lexical variables ‘phonological neighbourhood nouns’ and ‘word frequency nouns’ and the morphosyntactic variable ‘verb regularity’ were removed. Overall KMO statistics ranged from average (0.50 – 0.69) to good (> 0.70). All Bartlett’s tests of sphericity were significant (*p* < 0.001). This means that the correlation matrix of the variables was not an identity matrix. The data were thus suitable for PCA.

#### Conversation Data

Several principal components were extracted from the conversation data. Two principal components were extracted from the semantic variables, explaining 82% of the variance in the original semantic subset. These components are independent and should be compared between patients treated with cerebellar tumour surgery and their control speakers. The first component (C1; 42% of variance explained) included *all semantic psycholinguistic variables* for nouns. The second component (C2; 41% of the variance explained) consisted of *all semantic psycholinguistic variables* for verbs.

Three principal components were extracted from the lexical subset of variables, explaining 73% of the variance in the original dataset. The first principal component (C1; 29% of variance explained) included the psycholinguistic variables *age of acquisition* (nouns and verbs) and *phonological neighbourhood* (verbs). The second component (C2; 24% of variance explained) consisted of *lexical diversity* (nouns and verbs) and *word frequency* (nouns). The third component (C3) included the psycholinguistic variable *word frequency* (nouns and verbs). This component explained 20% of the data variance.

Three principal components were identified for the subset of morphosyntactic variables, explaining 83% of the variance in the original data. The first component (C1; 31% of variance explained) consisted of the *variable grammatical accuracy* and the *proportion of regular verbs*. The second component (C2; 27% of variance explained) and third component (C3; 25% of variance explained) each only consisted of one variable, respectively, *mean length of utterance* and the *proportion of transitive verbs*. The different components per level of language processing and the loadings of the variables per subset can be found in Table 3.

**Table 3:** Principal component structure and component loadings (> 0.45) after Varimax rotation for the subsets of variables in the cerebellar tumour group and control group for the conversation data. The principal components (C1, C2, C3) are ordered according to their importance in explaining the data variation with C1 explaining most variation. Variables in bold have a loading > 0.45 and thus make a significant contribution to a component

*Level of language processing*	*Variable*	*C1*	*C2*	*C3*
*Semantic*	Concreteness nouns	**0.96**	0.05	-
	Concreteness verbs	0.11	**0.95**	-
	Imageability nouns	**0.97**	0.08	-
	Imageability verbs	-0.05	**0.92**	-
	Verb instrumentality	0.44	**0.52**	-
*Lexical*	TTR nouns	0.10	**0.78**	-0.06
	TTR verbs	0.11	**0.83**	0.09
	AoA nouns	**0.85**	0.16	-0.04
	AoA verbs	**0.85**	0.19	-0.06
	Word frequency nouns	0.12	**-0.51**	**0.70**
	Word frequency verbs	-0.31	0.19	**0.81**
	Phonological neighbourhood verbs	**-0.67**	0.24	0.45
*Morphosyntactic*	MLU	-0.07	**0.95**	-0.07
	Grammatical accuracy	**0.88**	0.07	0.17
	Verb transitivity	0.05	-0.05	**0.98**
	Verb regularity	**0.68**	-0.41	-0.14

*C* = principal component; *TTR* = type-token ratio; *AoA* = age of acquisition; *MLU* = mean length of utterance.

#### Picture Descriptions

Several components were extracted from the picture descriptions in sentences. Two principal components were extracted from the semantic subset of variables, explaining 85% of the variance in the original semantic variables subset. The first component (C1; 48% of data variance explained) consisted of *all semantic psycholinguistic variables* for verbs. The second component (C2; 37% of variance explained) included *all semantic psycholinguistic variables* for nouns.

From the lexical subset of variables, three principal components were extracted, explaining 75% of the data variance. The first principal component (C1) explained 34% of the data and included *lexical accuracy* and the lexical psycholinguistic variables for verbs (i.e., *word frequency, age of acquisition and phonological neighbourhood*). The second component (C2; 23% of variance explained) consisted of *lexical diversity* for nouns and verbs. The third component (C3; 17% of variance explained) included the psycholinguistic variable *age of acquisition* (nouns).

Two principal components were identified for the subset of morphosyntactic variables, explaining 78% of the data variance. The first component (C1; 42% of variance explained) included *grammatical accuracy* and *finiteness index*. The second component (C2; 35% of variance explained) consisted of *mean length of utterance* and the *proportion of transitive verbs*. The different components and the individual loadings per subset of variables can be found in Table 4.

**Table 4:** Principal component structure and component loadings (> 0.45) after Varimax rotation for the subsets of variables in the cerebellar tumour and control group for the picture data. The principal components (C1, C2, C3) are ordered according to their importance in explaining the data variation with C1 explaining most variation. Variables in bold have a loading > 0.45 and thus make a significant contribution to a component

*Level of language processing*	*Variable*	*C1*	*C2*	*C3*
*Semantic*	Concreteness nouns	0.02	**0.94**	-
	Concreteness verbs	**0.94**	0.13	-
	Imageability nouns	0.08	**0.94**	-
	Imageability verbs	**0.94**	0.19	-
	Verb instrumentality	**0.81**	-0.13	-
*Lexical*	TTR nouns	0	**0.86**	0.16
	TTR verbs	-0.13	**0.87**	-0.11
	Lexical accuracy	**0.84**	0.04	-0.26
	AoA nouns	0.20	0.06	**0.89**
	AoA verbs	**0.82**	-0.05	0.35
	Word frequency verbs	**-0.67**	0.04	-0.32
	Phonological neighbourhood verbs	**-0.72**	0.28	-0.28
*Morphosyntactic*	MLU	0.22	**0.79**	-
	Grammatical accuracy	**0.92**	0.13	-
	Finiteness index	**0.90**	0.17	-
	Verb transitivity	-0.04	**-0.87**	-

*C* = principal component; *TTR* = type-token ratio; *AoA* = age of acquisition; *MLU* = mean length of utterance.

### Individual Case Statistics

The scores on the variables previously identified as principal components were compared for every patient who had cerebellar tumour surgery to five age- and gender-matched controls using Crawford’s modified t-tests. When describing the results, in addition to characterising the sample as a whole, a distinction was made between the patients who were diagnosed with pCMS (n = 5) and those who were not (n = 7). In total, nine out of twelve (75%) patients presented with an atypical spontaneous language profile in the conversational data and/or picture descriptions, suggesting a language impairment. Overall, three out of five patients in the pCMS-group had an atypical spontaneous language profile. More specifically, two patients (40%) showed evidence of a semantic deficit, one (20%) of lexical, three (60%) of morphosyntactic and one (20%) of phonological deficit. Six out of seven patients without pCMS had an atypical spontaneous language profile. Three (42.86%) presented evidence of semantic, two (28.57%) of lexical, three (42.86%) of morphosyntactic and four (57.14%) of phonological impairments. The raw scores (*z* values) for every patient and his/her controls and the results of the individual comparisons (*p* values) can be found in Tables 5, 6, 7, 8.

#### Conversation

**Fig. 1:**
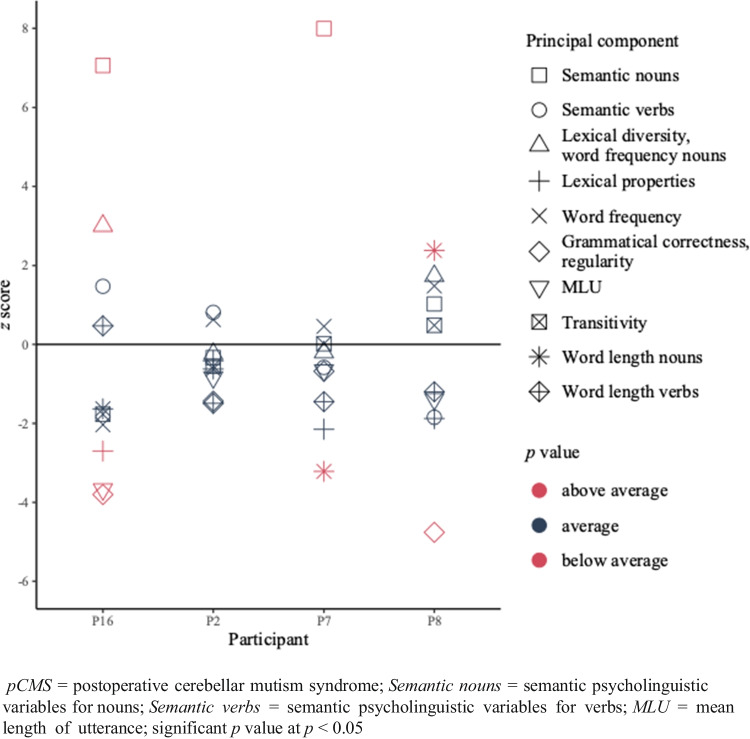
*z* scores of the patients who suffered from pCMS (n = 4) for the conversation

Of the pCMS-group, two (i.e., P7 and P16) out of four presented with a possible semantic deficit. P16 also presented evidence of a lexical impairment, as indicated by atypical scores on the lexical properties. Other patients in the pCMS-group did not have a lexical deficit. Two patients (i.e., P8 and P16) had a deviant score for one or more of the morphosyntactic components. Finally, one patient (i.e., P7) showed evidence of a phonological deficit. P8, on the other hand, scored better on the phonological variables compared to her controls. Other statistical comparisons between conversation data of patients who suffered from pCMS and corresponding controls did not yield significant differences. P2 scored within the average range for all measures. Two patients (i.e., P7 and P16) presented with multi-level impairments. In Fig. 1, the individual *z* scores of every patient for the conversation data can be found for every component. See Table 5 for results of individual comparisons.

**Table 5:** Individual raw scores for every measure and results of the comparisons for the pCMS-group (n = 4) and their controls for the conversation data

*Level of language processing*	*Measure*	*raw score*		*p value*	*raw score*		*p value*	*raw score*		*p value*	*raw score*		*p value*
		*P8*	*controls (M(SD))*		*P2*	*controls (M(SD))*		*P7*	*controls (M(SD))*		*P16*	*controls (M(SD))*	
Semantic	*Psycholinguistic variables nouns*	0,65	0,10 (0,54)	0,194	0,32	0,41 (0,27)	0,335	0,92	-1,34 (0,28)	0,001*	0,79	-1,37 (0,31)	0,001*
	*Psycholinguistic variables verbs*	-0,58	0,22 (0,43)	0,083	0,53	0,22 (0,38)	0,238	-0,43	0,07 (0,86)	0,289	1,28	-0,00 (0,87)	0,120
Lexical	*AoA, phonological neighbourhood verbs*	-1,63	-0,53 (0,59)	0,080	-0,67	-0,53 (0,33)	0,323	-0,07	1,44 (0,71)	0,060	-0,58	1,41 (0,74)	0,035*
	*Lexical diversity, word frequency nouns*	0,12	-0,88 (0,58)	0,093	-1,17	-1,08 (0,37)	0,350	-0,19	-0,13 (0,29)	0,354	1,14	0,04 (0,36)	0,026*
	*Word frequency*	1,20	-0,17 (0,92)	0,120	-0,45	-0,75 (0,47)	0,278	0,36	0,10 (0,59)	0,313	-1,26	0,04 (0,64)	0,068
Morphosyntactic	*Grammatical correctness, regularity*	-2,36	0,12 (0,52)	0,006*	-0,97	-0,22 (0,52)	0,127	0,01	0,41 (0,58)	0,265	-1,13	0,46 (0,42)	0,013*
	*MLU*	-0,25	0,85 (0,79)	0,132	0,15	0,76 (0,73)	0,231	0,31	0,87 (0,85)	0,271	-2,30	0,94 (0,88)	0,014*
	*Transitivity*	0,53	0,07 (0,95)	0,307	-0,73	-0,17 (1,01)	0,294	-0,56	-0,57 (0,49)	0,366	-1,45	-0,57 (0,50)	0,091
Phonological	*Word length nouns*	5,54	4,85 (0,29)	0,049*	4,76	4,94 (0,28)	0,278	4,89	5,92 (0,32)	0,021*	5,50	6,02 (0,32)	0,104
	*Word length verbs*	4,67	5,30 (0,53)	0,164	5,00	5,46 (0,30)	0,119	5,33	5,84 (0,35)	0,124	6,00	5,83 (0,35)	0,312

*AoA* = age of acquisition; *MLU* = mean length of utterance; * = significant difference (*p* value < 0.05)

**Fig. 2:**
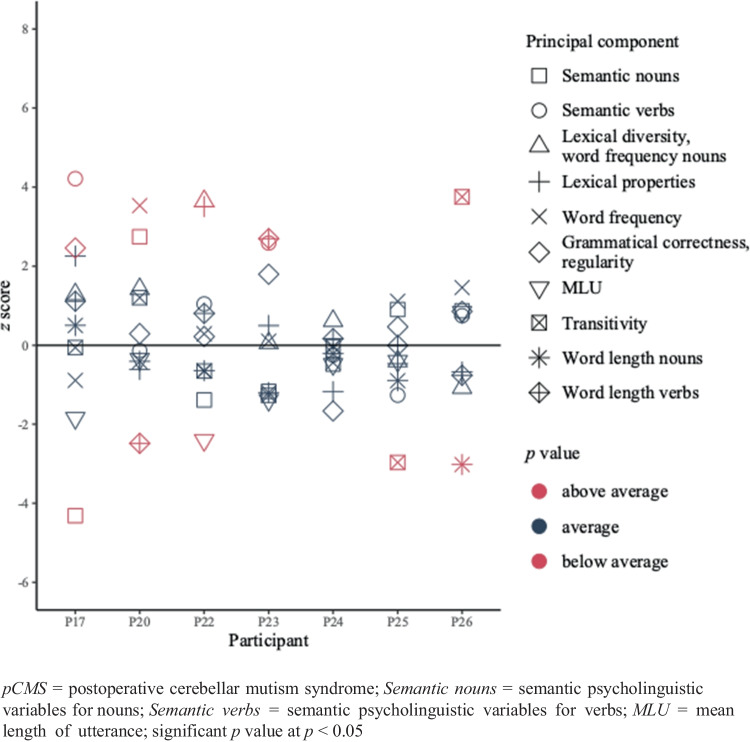
*z* scores of the patients who did not experience pCMS (n = 7) for the conversation data

 Of the seven patients who did not experience pCMS, three (i.e., P17, P20 and P23) showed evidence of a semantic processing impairment (nouns or verbs). P17 had deviant scores for the semantic verb component but scored better than his controls for noun semantic processing. Two patients (i.e., P20 and P22) had deviant scores for one or more of the included lexical components. Another two patients (i.e., P22 and P25) showed evidence of a morphosyntactic deficit. P17 and P26, on the other hand, scored better compared to their controls for one or more of the morphosyntactic variables. Finally, P26 and P20 scored below average for word length, indicating a phonological deficit. P24 scored within the normal range for all measures. Other statistical comparisons between conversation data of patients who did not experience pCMS and corresponding controls did not yield significant differences. Two participants (i.e., P20, P22) presented with multi-level impairments. See Fig. 2 for the individual *z* scores for every component and Table 6 for individual *p* values.

**Table 6:** Individual raw scores for every measure and results of the comparisons for the non-pCMS-group (n = 7) and their controls for the conversation data

*Level of language processing*	*Measure*	*raw score*		*p value*	*raw score*		*p value*	*raw score*		*p value*	*raw score*		*p value*
		*P17*	*controls (M(SD))*		*P25*	*controls (M(SD))*		*P24*	*controls (M(SD))*		*P20*	*controls (M(SD))*	
Semantic	*Psycholinguistic variables nouns*	0,06	1,23 (0,27)	0,009*	1,68	0,91 (0,86)	0,219	0,60	0,69 (0,18)	0,310	1,02	0,39 (0,23)	0,032*
	*Psycholinguistic variables verbs*	2,31	-0,69 (0,71)	0,010*	-0,14	0,84 (0,78)	0,150	-0,56	-0,32 (0,96)	0,348	-0,68	-0,56 (0,88)	0,360
Lexical	*AoA, phonological neighbourhood verbs*	-0,57	-1,09 (0,23)	0,053	-0,62	-0,33 (0,60)	0,310	-1,19	-0,32 (0,74)	0,167	-0,25	0,16 (0,66)	0,281
	*Lexical diversity, word frequency nouns*	-0,42	-1,02 (0,47)	0,149	-0,21	0,12 (0,81)	0,324	1,07	0,57 (0,82)	0,281	1,09	0,07 (0,72)	0,131
	*Word frequency *	-0,38	0,61 (1,12)	0,220	1,75	-0,08 (1,65)	0,179	-0,52	-0,08 (1,02)	0,318	1,54	-0,75 (0,65)	0,016*
Morphosyntactic	*Grammatical correctness, regularity*	0,39	-1,18 (0,64)	0,044*	0,88	0,03 (1,82)	0,312	-1,05	0,31 (0,82)	0,102	0,49	0,11 (1,30)	0,341
	*MLU*	-2,36	-1,17 (0,64)	0,082	-0,98	-0,59 (0,98)	0,326	-0,48	-0,07 (0,86)	0,308	-0,49	-0,24 (0,70)	0,332
	*Transitivity *	0,15	0,20 (1,01)	0,366	-1,90	0,64 (0,86)	0,026*	-0,03	0,01 (1,25)	0,366	1,58	0,66 (0,76)	0,166
Phonological	*Word length nouns*	4,67	4,48 (0,38)	0,304	4,60	5,35 (0,84)	0,219	4,83	5,02 (0,87)	0,354	4,82	5,06 (0,59)	0,324
	*Word length verbs*	5,29	5,01 (0,25)	0,179	5,29	5,29 (0,58)	0,365	5,31	5,25 (0,38)	0,358	4,86	5,82 (0,39)	0,042*

*Level of language processing*	*Measure*	*raw score*		*p value*	*raw score*		*p value*	*raw score*		*p value*
		*P23*	*controls (M(SD))*		*P26*	*controls (M(SD))*		*P22*	*controls (M(SD))*	
Semantic	*Psycholinguistic variables nouns*	-1,37	-0,29 (0,92)	0,169	0,46	-0,39 (0,98)	0,224	-1,52	-1,06 (0,33)	0,135
	*Psycholinguistic variables verbs*	3,05	-0,79 (1,48)	0,039*	0,65	-0,25 (1,20)	0,252	0,64	0,04 (0,57)	0,190
Lexical	*AoA, phonological neighbourhood verbs*	0,47	-0,02 (0,99)	0,305	-0,72	-0,31 (0,61)	0,265	2,60	0,76 (0,52)	0,016*
	*Lexical diversity, word frequency nouns*	1,44	1,39 (1,07)	0,365	0,24	1,18 (0,88)	0,184	0,51	-0,97 (0,41)	0,014*
	*Word frequency *	0,43	0,29 (1,35)	0,363	1,73	-0,05 (1,22)	0,125	0,14	-0,08 (0,73)	0,344
Morphosyntactic	*Grammatical correctness, regularity*	1,97	0,23 (0,97)	0,089	1,04	0,05 (1,16)	0,227	0,00	-0,16 (0,75)	0,352
	*MLU*	-1,05	-0,48 (0,41)	0,131	0,07	-0,28 (0,41)	0,232	0,20	0,85 (0,27)	0,048
	*Transitivity *	-1,22	0,39 (1,27)	0,153	2,81	-0,14 (0,78)	0,014*	-0,74	-0,22 (0,81)	0,272
Phonological	*Word length nouns*	4,50	6,10 (1,33)	0,164	4,38	6,19 (0,60)	0,025*	5,43	5,63 (0,31)	0,273
	*Word length verbs*	6,13	5,35 (0,29)	0,035*	5,00	5,38 (0,50)	0,249	6,07	5,75 (0,40)	0,241

*AoA* = age of acquisition; *MLU* = mean length of utterance; * = significant difference (*p* value < 0.05)

#### Picture descriptions

**Fig. 3:**
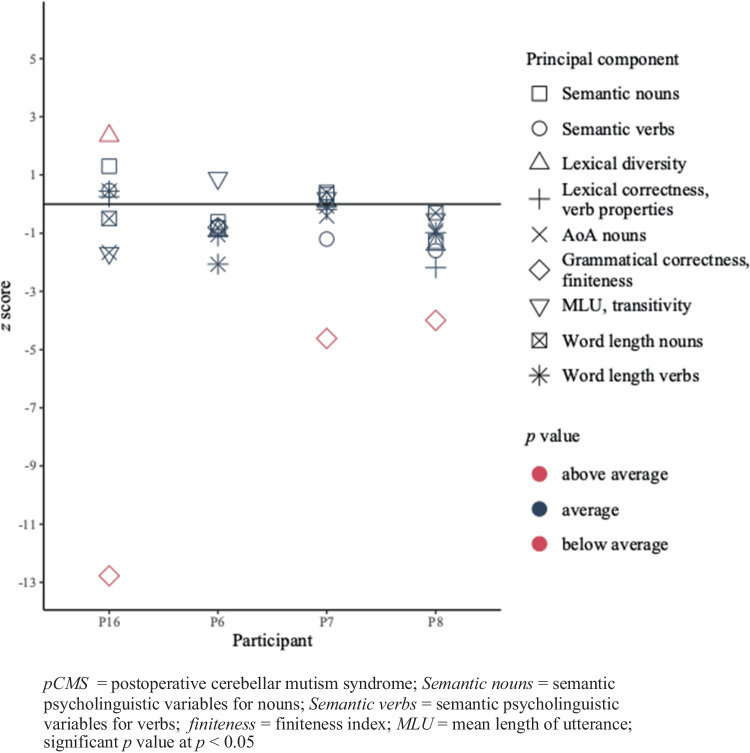
*z* scores of the participants who were diagnosed with pCMS (n = 4) for the picture description data

For the picture descriptions in sentences, patients who experienced pCMS did not score significantly different compared to controls on semantic components. P16 performed better than his controls for lexical diversity. Three (i.e., P7, P8, P16) out of four patients had a possible morphosyntactic impairment. None of the patients had deviant scores on the phonological components. P6 scored within the average range for all measures. Other statistical comparisons between picture description data of the pCMS-group and corresponding controls did not yield significant differences. None of the patients presented with multi-level impairments. The individual *z* scores for every principal component can be found in Fig. 3 (*p* values in Table 7).

**Table 7:** Individual raw scores for every measure and results of the comparisons for the pCMS-group (n = 4) and their controls for the picture description data

*Level of language processing*	*Measure*	*raw score*		*p value*	*raw score*		*p value*	*raw score*		*p value*	*raw score*		*p value*
		*P6*	*controls (M(SD))*		*P8*	*controls (M(SD))*		*P7*	*controls (M(SD))*		*P16*	*controls (M(SD))*	
Semantic	*Psycholinguistic variables nouns*	-0,05	0,43 (0,80)	0,282	-0,12	0,50 (0,49)	0,149	-0,70	-1,02 (1,01)	0,337	0,29	-1,02 (1,01)	0,148
	*Psycholinguistic variables verbs*	-0,94	0,48 (1,85)	0,247	-0,75	0,02 (0,48)	0,107	-0,84	0,33 (0,98)	0,162	0,79	0,33 (0,98)	0,310
Lexical	*Lexical correctness, verb properties*	-1,13	0,00 (1,03)	0,183	-1,13	0,27 (0,64)	0,058	0,54	0,72 (1,03)	0,355	0,98	0,72 (1,03)	0,349
	*Lexical diversity*	-0,05	0,78 (0,89)	0,215	-1,39	0,29 (1,21)	0,130	-0,26	-0,33 (0,73)	0,363	1,37	-0,33 (0,73)	0,048*
	*AoA nouns*	-1,33	-0,59 (0,71)	0,190	-1,13	-0,34 (0,87)	0,217	1,00	1,32 (0,78)	0,321	0,02	1,32 (0,78)	0,098
Morphosyntactic	*Grammatical correctness, finiteness index*	-1,26	-0,34 (1,15)	0,242	-2,02	0,68 (0,68)	0,011*	-0,79	0,43 (0,26)	0,007*	-2,95	0,43 (0,26)	< 0.001*
	*MLU, transitivity*	0,13	-1,11 (1,39)	0,219	-0,15	0,20 (0,66)	0,299	0,96	0,86 (0,53)	0,355	-0,05	0,86 (0,53)	0,098
Phonological	*Word length nouns*	4,95	5,25 (0,37)	0,238	4,93	5,11 (0,60)	0,342	5,87	5,74 (0,33)	0,325	5,58	5,74 (0,33)	0,307
	*Word length verbs*	5,05	5,43 (0,19)	0,067	5,21	5,69 (0,50)	0,201	5,76	5,78 (0,33)	0,366	5,93	5,78 (0,33)	0,316

*AoA* = age of acquisition; *MLU* = mean length of utterance; * = significant difference (*p* value < 0.05)

**Fig. 4:**
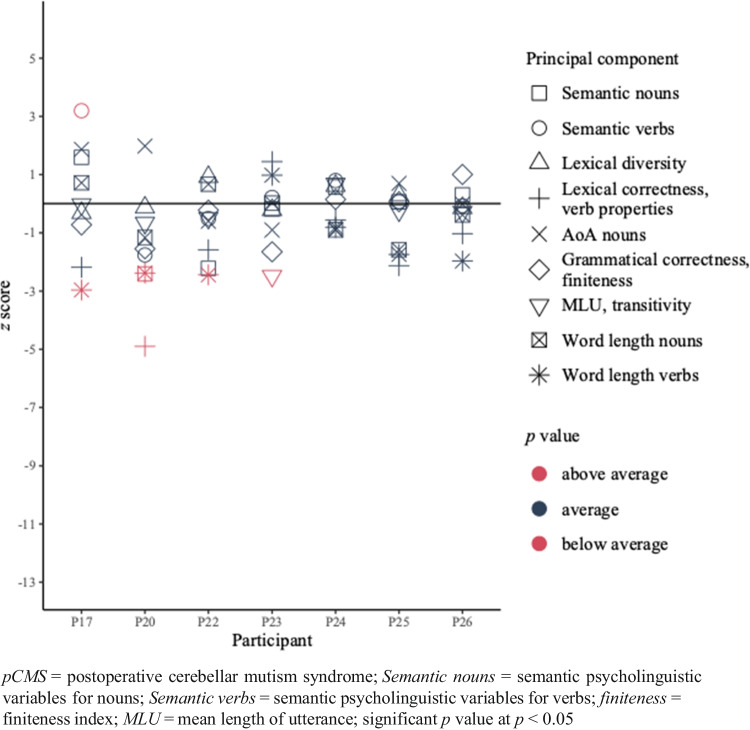
*z* scores of the patients who did not experience pCMS (n = 7) for the picture description data

 Of the seven patients in the non-pCMS-group, only P17 had scores indicative of a semantic impairment, while P20 scored better for semantic processing compared to his controls. P20 also had a lower score on one of the lexical components. One out of seven patients (i.e., P23) had a possible morphosyntactic deficit. Finally, three patients (i.e., P17, P20 and P22) scored lower compared to their controls for word length, indicating a phonological deficit. P24, P25 and P26 showed no significant differences compared to their controls (see Fig. 4). Other statistical comparisons between picture description data of patients who did not experience pCMS and corresponding controls did not yield significant differences. Two patients (i.e., P17 and P20) presented with multi-level impairments. The individual *z* scores for every principal component can be found in Fig. 4. Results of the individual comparisons between patients and their controls can be found in Table 8.

**Table 8:** Individual raw scores for every measure and results of the comparisons for the non-pCMS-group (n = 7) and their controls for the picture description data

*Level of language processing*	*Measure*	*raw score*		*p value*	*raw score*		*p value*	*raw score*		*p value*	*raw score*		*p value*
		*P17*	*controls (M(SD))*		*P25*	*controls (M(SD))*		*P24*	*controls (M(SD))*		*P20*	*controls (M(SD))*	
Semantic	*Psycholinguistic variables nouns*	1,86	0,92 (0,59)	0,108	0,48	0,43 (0,80)	0,367	0,39	0,09 (0,54)	0,290	-2,12	0,15 (0,94)	0,046*
	*Psycholinguistic variables verbs*	-0,05	-1,13 (0,34)	0,022*	0,61	0,48 (1,85)	0,364	1,02	0,46 (0,71)	0,242	-1,86	-0,56 (0,74)	0,090
Lexical	*Lexical correctness, verb properties*	-2,43	-0,67 (0,81)	0,057	-2,20	0,00 (1,03)	0,061	-0,35	0,10 (0,80)	0,291	-2,39	0,15 (0,52)	0,005*
	*Lexical diversity*	-1,28	-0,78 (1,55)	0,342	1,04	0,78 (0,89)	0,344	0,40	-0,12 (0,85)	0,280	0,19	0,30 (0,89)	0,361
	*AoA nouns*	0,14	-1,58 (0,92)	0,080	-0,09	-0,59 (0,71)	0,261	-0,03	0,25 (0,31)	0,222	1,51	-0,01 (0,77)	0,072
Morphosyntactic	*Grammatical correctness, finiteness index*	-0,81	-0,19 (0,85)	0,257	-0,26	-0,34 (1,15)	0,366	0,31	0,10 (1,41)	0,360	-1,20	0,03 (0,79)	0,113
	*MLU, transitivity*	-0,86	-0,84 (1,00)	0,365	-1,44	-1,11 (1,39)	0,350	0,47	-0,25 (1,06)	0,267	-0,79	-0,12 (1,02)	0,267
Phonological	*Word length nouns*	5,14	4,82 (0,45)	0,260	4,67	5,25 (0,37)	0,109	4,87	5,33 (0,51)	0,220	4,86	5,59 (0,63)	0,170
	*Word length verbs*	4,50	5,14 (0,22)	0,027*	5,11	5,43 (0,19)	0,093	5,53	5,77 (0,30)	0,238	4,82	5,47 (0,27)	0,047*

*Level of language processing*	*Component*	*raw score*		*p value*	*raw score*		*p value*	*raw score*		*p value*
		*P23*	*controls (M(SD))*		*P26*	*controls (M(SD))*		*P22*	*controls (M(SD))*	
Semantic	*Psycholinguistic variables nouns*	0,23	0,33 (0,55)	0,356	0,50	0,33 (0,55)	0,343	-2,51	-1,05 (0,66)	0,057
	*Psycholinguistic variables verbs*	0,58	0,41 (0,81)	0,353	0,35	0,41 (0,81)	0,366	-0,26	0,26 (1,01)	0,303
Lexical	*Lexical correctness, verb properties*	0,87	0,09 (0,54)	0,126	-0,47	0,09 (0,54)	0,194	-0,11	0,90 (0,64)	0,110
	*Lexical diversity*	-0,42	-0,11 (1,36)	0,352	-0,32	-0,11 (1,36)	0,360	0,46	0,02 (0,48)	0,215
	*AoA nouns*	-0,20	0,49 (0,76)	0,217	0,48	0,49 (0,76)	0,366	-0,03	0,38 (0,67)	0,280
Morphosyntactic	*Grammatical correctness, finiteness index*	-0,92	0,22 (0,69)	0,103	0,91	0,22 (0,69)	0,198	0,63	0,74 (0,50)	0,350
	*MLU, transitivity*	0,17	0,91 (0,30)	0,042*	0,82	0,91 (0,30)	0,344	0,12	0,47 (0,70)	0,306
Phonological	*Word length nouns*	5,13	5,12 (0,25)	0,366	5,02	5,12 (0,25)	0,328	5,83	5,51 (0,47)	0,267
	*Word length verbs*	5,88	5,69 (0,19)	0,203	5,31	5,69 (0,19)	0,072	5,35	5,84 (0,20)	0,044*

*AoA* = age of acquisition; *MLU* = mean length of utterance; * = significant difference (*p* value < 0.05)

## Discussion

In the present study, we aimed to (1) identify variables extracted from spontaneous language which differentiate between patients treated with cerebellar tumour surgery and healthy controls and (2) characterise the spontaneous language outcomes of twelve patients treated with cerebellar tumour surgery. This is the most comprehensive spontaneous language analysis in this population up to date by including four levels of language processing. Nine out of twelve patients showed evidence of a language impairment as reflected by an atypical spontaneous language profile (i.e., the patient scored significantly different compared to his or her controls for one or more of the included variables). These were persistent impairments, affecting language in the long-term, as patients were on average four years after surgery at the time of language assessment.

### Principal Components and the Potential to Differentiate Groups Based On a Psycholinguistic Approach to Spontaneous Language Analysis

Results from the PCA indicate that the variables used in individual case–control comparisons do have the potential to differentiate these groups, as each component explained a substantial amount of variance. Within each level of processing and separately for the conversation and picture data, the PCA produced independent components which clearly reflect different aspects of processing within a level. For example, in the picture data the PCA divided the semantic psycholinguistic variables into a noun and verb component, possibly indicating that these should be considered separately in our population. This is in line with previous research in a variety of clinical populations indicating that nouns and verbs can be selectively impaired [74, 75]. Nouns were also separated in the identified morphosyntactic components, but the majority of the morphosyntactic variables were verb specific. This differentiation in the PCA between noun- and verb-related variables cannot be studied in a controlled manner within our spontaneous language data; however, the results indicate that this should be explored in future research aiming to test dissociations between nouns and verbs in children treated with cerebellar tumour surgery.

Another clinically relevant observation from the PCA data is that psycholinguistic spontaneous language variables were often clustered together in the PCA and cannot be seen as independent from each other. Likewise, the PCA clustered some psycholinguistic variables together with standard language measures in a component (e.g., lexical accuracy with frequency, AoA, and phonological neighbourhood for verbs in the picture data). Such results have methodological implications for studies in which each variable is compared independently between groups.

Several psycholinguistic variables, such as phonological neighbourhood (nouns) and verb regularity (in picture data) were removed from the PCA as they did not contribute to the data variance. Such variables were not included in further case–control comparisons, thus taking advantage of PCA’s potential to aid in dimensionality reduction [76]. This way, PCA worked as a preparatory step for individual case–control comparisons, which are discussed next.

### Characterising Language Impairments at the Individual Level

The results of this study showed spontaneous language impairments across all levels. Comparisons involving semantic variables detected impairments in five out of twelve patients. These were identified with the psycholinguistic noun and verb component. This is a unique contribution of the present study, as semantic processing has not been distinguished from lexical processing in previous studies, even though cerebellar involvement in semantic processing has been reported in non-neurological-impaired individuals [51, 52]. At the lexical level, three out of twelve patients showed impairments which were detected across all included lexical variables (e.g., lexical correctness, AoA, word frequency). Comparisons involving morphosyntactic variables detected impairments in six out of twelve patients. Again, impairments were reported across all morphosyntactic variables. Five out of twelve patients had a possible phonological impairment which was detected by the psycholinguistic variable word length (nouns and verbs). Previous studies also reported lexical-semantic, phonological and morphosyntactic impairments in a comparable clinical population [7, 11, 12, 16].

The present study also investigated the differential spontaneous language outcomes in patients who were diagnosed with pCMS and those who were not. Similar to previous studies [11, 16] most patients in our study presented with long-term atypical spontaneous language profiles irrespective of a previous pCMS diagnosis. Even though the sample size was small, the nature of the suggested impairments appears to differ somewhat between groups. Three out of five patients who suffered from pCMS had a deviant score on predominantly semantic (i.e., ‘semantic psycholinguistic variables nouns’) and morphosyntactic (i.e., ‘grammatical accuracy’ and ‘mean length of utterance’) components. Other language processing impairments were also observed for the conversation data, but only morphosyntactic impairments were present in the picture descriptions. Furthermore, two patients (P2 and P6) had a severe reduction of self-generated language, limiting spontaneous language analysis as reported in a previous study by Riva and Giorgi [11]. These deficits could be a possible long-term adverse effect of pCMS.

Semantic and morphosyntactic impairments were also observed in the non-pCMS-group, but the language impairments seemed to encompass different language processing levels more broadly than in the pCMS-group (where morphosyntactic deficits were more frequent than other deficits). Furthermore, this group also had a similar incidence of lexical and phonological deficits in both the conversation and picture description data. The two previous studies comparing patients with and without pCMS did not report differences in the nature of the observed spontaneous language deficits [11, 16]. However, in Cámara et al. [7] worse verbal memory outcomes were found during formal testing in children who suffered from pCMS. Furthermore, children in the pCMS-group had additional lexical-semantic impairments that were not observed in the non-pCMS group [7]. Differently, in the present study, the tendency to produce shorter words (which can be indicative of worse verbal memory) was identified in patients of both groups as were lexical-semantic impairments. This highlights the importance of reporting individual case–control comparisons, in addition to group comparisons, to document deficits that were not identified on a group level.

While we discussed possible differences between groups, these results need to be interpreted with caution given the small and unequal sample sizes. Although there were tendencies towards differences in the nature of the deficits, the same impairments or the same severity of impairments were not observed in all patients belonging to a group. Replications in bigger and equal patient groups will be better placed to answer questions of prevalence.

In the pCMS-group a high incidence of morphosyntactic deficits in conversation and picture descriptions was observed. Studies by, for example, Di Rocco et al. [23] suggested preoperative lexical-semantic and/or morphosyntactic impairments to be a possible risk factor for pCMS. In our study, language impairments and a reduced spontaneous language output were still observable after the mutism resolved and were not found in patients who did not experience pCMS. This is in line with earlier studies [23, 54]. It should be further investigated if morphosyntactic impairments in pCMS-patients indeed originate pre-operatively as a result of tumour growth or presence. Differently, the linguistic deficits in the non-pCMS group might be more intrinsically linked to cerebellar lesions which occur during tumour surgery.

While morphosyntactic impairments have often been reported as part of the cerebellar cognitive-affective syndrome (CCAS), they have scarcely been reported in relation to pCMS [7, 16, 77]. Our results might suggest that pCMS should be regarded as a hyper-acute severe form of CCAS, rather than a separate clinical syndrome [78, 79]. It is also possible that the physiopathological mechanisms underlying the atypical spontaneous language profiles differ between groups. Yet, no preoperative language assessment was conducted and no diffusion tensor imaging data were available for our patient group. In order to test these hypotheses, future research would need to compare the pre- and postoperative spontaneous language outcomes in patients with and without pCMS, and relate these outcomes to the integrity of the cerebello-cerebral circuitry by means of MRI studies [80–82].

Even though our results suggest differential patterns of spontaneous language outcomes in the pCMS- and non-pCMS-group, interindividual heterogeneity was still observed both in the nature and the severity of the deficits, as reported in earlier research [72]. This finding might be explained by several variables related to the cerebellar tumour survivor (e.g., age at surgery [83]), the tumour (e.g., location or presence of a hydrocephalus [7]) or treatment (e.g., neurotoxicity caused by radiotherapy [11]). In our patient group, however, no obvious relation was found between these variables and the spontaneous language outcomes. This might be attributed to the limited size and large diversity of our clinical sample.

On the other hand, the severity of the language deficits (i.e., the number of impaired variables and/or the degree to which individual variables were impaired) in patients who experienced pCMS has also been related to the length of the mute phase [6]. This also seemed to be the case in our pCMS-group, with patients who were mute for several months following surgery (i.e., P7 and P16) presenting with severe multi-level impairments as opposed to other patients (e.g., P2) who were mute for a shorter period. It is possible that these more severe language impairments are caused by a reduced language experience or the influence of dysarthria during language acquisition. Nonetheless, P7 and P16 were already teenagers at the time of surgery, when language acquisition is near-complete.

Finally, even though the present study was limited to a linguistic analysis, the possible influence of cognitive impairment on the language outcomes cannot be ignored. For example, working memory has been reported to influence word learning and sentence processing which are important for language development [84, 85]. Cerebellar tumour survivors can present with a variety of postoperative cognitive impairments (e.g., working memory problems, attentional deficits; see Wolfe et al. for a review [86]) and these might have contributed to the observed heterogeneity. Unfortunately, it was not possible to investigate the influence of cognitive impairment in the current sample because of the inconsistent (in time and targeted functions) neuropsychological assessments across individuals, but this should be explored in future studies. The present results do confirm that cerebellar tumour survivors have a broad spectrum of long-term language impairments, as was reported in previous studies [7, 16]. These may significantly hinder daily communication years after surgery and should be assessed comprehensively in clinical practice [18].

### Methodological Considerations

Since this study introduced a novel method of spontaneous language analysis, some methodological remarks can be made. The present study showed that PCA can be a promising aid when interpreting patterns of impairment in a heterogeneous clinical population. Future studies should explore if the same principal components are identified in a similar patient group. It should be mentioned that, even though we counted the included spontaneous language variables separately and attributed them to separate language processing levels, these variables, levels, and impairments at a given level interact during language production [87]. For example, when producing a sentence, a semantic processing deficit might induce a highly concrete (i.e., less semantically rich) verb, but also an intransitive (i.e., less syntactically complex) verb due to higher processing demands. The interactions could provide (at least in part) an explanation for the observed heterogeneity in our sample.

Overall, both standard variables (e.g., grammatical accuracy, MLU) and psycholinguistic variables (e.g., imageability, word length) could differentiate individual patients from their controls. Several standard variables identified lexical-semantic, phonological and morphosyntactic impairments in our patient group, in accordance with previous studies [7, 11, 12, 16]. Nonetheless, analysing the psycholinguistic variables allowed us to characterise the nature of the observed deficits in more detail than possible via standard spontaneous language measures and structured tasks used in earlier studies [30]. Our results suggest that semantic and phonological psycholinguistic variables can identify language processing problems that are not found with standard spontaneous language measures (e.g., lexical diversity). However, not all psycholinguistic variables could be used to identify spontaneous language deficits. For example, scores on standard morphosyntactic measures (e.g., grammatical accuracy) suggested language impairments that were not identified with morphological and syntactic properties (e.g., verb transitivity and regularity). In P25, on the other hand, a morphosyntactic impairment was evident with the word property ‘transitivity’ that was not identified with standard morphosyntactic measures. Regarding verb regularity, the generalisability of our findings might be limited to languages that share linguistic properties with Dutch (e.g., English, German [88, 89]).

Interestingly, fewer atypical spontaneous language profiles were found for the picture descriptions than for the conversation. Although both elicitation methods require the integration of different levels of linguistic processing, the visual representation of objects or actions in pictures limits the number of possible verbal responses and the possible variety in several psycholinguistic properties [21]. For example, all presented nouns in the pictures were highly concrete. This might explain why lexical-semantic processing deficits were only identified in spontaneous conversation. Finally, two of the three administered pictures were meant for language assessment in children, while several patients were adults at follow-up [65]. Therefore, picture description tasks might have been easier than spontaneous conversation.

In what concerns the feasibility of using this approach in clinical practice it should also be noted that, while gathering a spontaneous language sample with the patient may not take long, and our results suggest that this method is able to uncover difficulties, extracting psycholinguistic properties from the produced nouns and verbs in spontaneous language is time consuming. Ratings for different psycholinguistic variables are spread out across databases and some values need to be calculated manually. Therefore, after careful validation of our method, the development of a user-friendly clinical tool is warranted.

Finally, it is important to note some limitations of the present study. Although the results suggest long-term spontaneous language impairments in our patient group, these should be interpreted with caution. The large diversity (e.g., regarding age range) and small sample size of our patient group make it difficult to draw definite conclusions regarding the long-term language outcomes in cerebellar tumour survivors. Therefore, future studies should replicate our methods and results in larger patient groups. These studies should also include more homogeneous subgroups of patients, regarding, for example, age and tumour type to further evaluate the possible influence of risk factors on language in this clinical population.

## Conclusions

In the present study, the long-term spontaneous language outcomes in participants who underwent cerebellar tumour surgery during childhood were investigated. Results show that many cerebellar tumour survivors have atypical spontaneous language profiles, suggesting language impairments. This was the first study to use psycholinguistic variables for a comprehensive spontaneous language assessment, showing promising results in terms of this tool being able to identify difficulties. For example, our method allowed us to identify isolated semantic and phonological impairments in spontaneous language that were not reported in previous studies. Possible differential patterns in the language outcomes in patients who were and were not diagnosed with pCMS were identified, implying different mechanisms underlying these impairments. Results show that spontaneous language might be a useful clinical assessment tool, allowing a short assessment time which is advantageous for patients who can become easily tired. Further research is necessary, however, to confirm these results and validate this novel method of spontaneous language analysis. A comprehensive postoperative language assessment in this clinical population is necessary irrespective of pCMS diagnosis, given the need for early intervention and the identification of language impairments across all levels of language processing.

### Supplementary Information

Below is the link to the electronic supplementary material.

Supplementary file1 (DOCX 38 KB)

## Data Availability

The patient data analysed during the current study were shared with us and requests for data sharing should go to Prof Philippe Paquier. Control data are available from the corresponding author on reasonable request and can be shared via DataverseNL.

## Abbreviations

AoA: Age of acquisition
ASTA: Analysis of Spontaneous Language in Aphasia
BI: Bicaudate index
CCAS: Cerebellar cognitive-affective syndrome
CCC: Children’s Communication Checklist
CELF: Clinical Evaluation of Language Fundamentals
CETO: Research Ethics Committee, Faculty of Arts University of Groningen
DutchPOND: Dutch interface of the Cross-Linguistic Easy-Access Resource for Phonological and Orthographic Neighbourhood Densities
KMO: Kaiser-Meyer-Olkin Measure of Sampling Adequacy
MLU: Mean length of utterance
MRI: Magnetic Resonance Imaging
PCA: Principal component analysis
pCMS: Postoperative cerebellar mutism syndrome
STAP: Spontaneous Language Analysis Procedure

## Footnotes

1 A corpus or text corpus is a large collection of written or spoken texts that are stored electronically. For example, the iWeb corpus contains fourteen billion English words extracted from twenty-two million web pages [90].

2 PCA is a data-driven approach. It generates a single multi-dimensional model based on the patterns of a group while capturing individual differences between participants [76, 91]. In other words, a large number of variables are summarized in a smaller number of inter-correlated quantitative variables or ‘emergent principal components’ that explain most of the data variance. Among others, PCA can be employed as a dimensionality reduction technique [76]. The variables that explain the least of the data variance (i.e., the noise in the data) are removed from the data. PCA has been employed in studies with adults with post-stroke aphasia [92, 93], but also in one study investigating the neuropsychological outcomes in children with a medulloblastoma [94].
