# Supplementary material for: Characterising the Long-Term Language Impairments of Children Following Cerebellar Tumour Surgery by Extracting Psycholinguistic Properties from Spontaneous Language
Source: Cerebellum. 2023 May 15;23(2):523–44. doi: 10.1007/s12311-023-01563-z (PMC10951034; doi:10.1007/s12311-023-01563-z)
Supplement: Supplementary file 1 — Supplementary file1 (DOCX 38 KB) [file 12311_2023_1563_MOESM1_ESM.docx]

**Supplementary Materials**

**Table 1** Individual scores for every psycholinguistic variable for the pCMS-group (n = 4) and their controls for the conversation data.

| ***Level of language processing*** | ***Psycholinguistic variable*** | ***P8 (M(SD))*** | ***controls (M(SD))*** | ***P2 (M(SD))*** | ***controls (M(SD))*** | ***P7 (M(SD))*** | ***controls (M(SD))*** | ***P16 (M(SD))*** | ***controls (M(SD))*** |
| --- | --- | --- | --- | --- | --- | --- | --- | --- | --- |
| *Semantic* | *Imageability nouns* | 6.04 (0.81) | 5.47 (1.31) | 5.76 (1.23) | 5.69 (1.24) | 5.98 (1.03) | 4.52 (1.40) | 5.95 (0.76) | 4.46 (1.39) |
|  | *Imageability verbs* | 4.49 (1.44) | 4.68 (1.44) | 4.93 (1.36) | 4.68 (1.42) | 4.49 (1.39) | 4.62 (1.36) | 5.01 (1.34) | 4.60 (1.35) |
|  | *Concreteness nouns* | 3.81 (1.06) | 3.59 (1.09) | 3.73 (0.91) | 3.75 (1.06) | 3.93 (1.04) | 2.75 (0.99) | 3.97 (0.63) | 2.74 (1.00) |
|  | *Concreteness verbs* | 2.71 (1.02) | 2.83 (1.04) | 2.84 (1.01) | 2.86 (0.99) | 2.66 (1.10) | 2.72 (0.89) | 3.09 (0.95) | 2.69 (0.89) |
|  | *Verb instrumentality** | 0.00 | 10.38 (5.45) | 8.70 | 10.52 (4.24) | 13.33 | 8.13 (5.51) | 21.43 | 8.26 (5.43) |
| *Lexical* | *AoA nouns* | 5.52 (0.99) | 5.62 (1.17) | 5.35 (2.02) | 5.44 (1.39) | 6.00 (1.88) | 7.67 (2.90) | 5.66 (1.37) | 7.79 (2.95) |
|  | *AoA verbs* | 4.88 (0.92) | 5.11 (0.95) | 5.12 (0.78) | 5.30 (1.13) | 5.09 (0.80) | 5.91 (1.73) | 5.11 (0.95) | 5.87 (1.66) |
|  | *Word frequency nouns* | 161.37 (275.22) | 176.25 (268.51) | 183.94 (216.52) | 162.22 (236.41) | 196.42 (251.59) | 185.49 (264.32) | 127.24 (95.48) | 174.54 (254.61) |
|  | *Word frequency verbs* | 2025.16 (2475.64) | 1357.27 (1992.19) | 1179.60 (1760.67) | 1088.49 (1707.23) | 1581.52 (2074.66) | 1160.97 (1929.26) | 754.99 (962.54) | 1185.05 (1944.96) |
|  | *Phonological neighbourhood nouns* | 6.82 (7.73) | 10.37 (9.02) | 8.84 (8.99) | 9.92 (8.93) | 12.71 (8.16) | 8.22 (8.22) | 8.00 (9.90) | 7.88 (8.10) |
|  | *Phonological neighbourhood verbs* | 15.11 (5.33) | 10.57 (6.40) | 10.83 (6.14) | 10.74 (6.90) | 9.53 (6.61) | 9.28 (6.81) | 10.46 (5.43) | 9.30 (6.85) |
| *Phonological* | *Word length nouns* | 5.54 (2.37) | 4.85 (2.18) | 4.76 (2.00) | 4.94 (2.26) | 4.89 (3.30) | 5.92 (2.80) | 5.50 (2.37) | 6.02 (2.84) |
|  | *Word length verbs* | 4.67 (1.00) | 5.30 (1.49) | 5.00 (1.28) | 5.46 (1.58) | 5.33 (2.29) | 5.84 (1.88) | 6.00 (2.08) | 5.83 (1.89) |
| *Morphosyntactic* | *Verb regularity** | 0.00 | 21.17 (7.55) | 17.39 | 21.76 (8.39) | 26.67 | 35.18 (8.16) | 50.00 | 34.75 (8.46) |
|  | *Verb transitivity** | 55.56 | 52.95 (12.60) | 43.48 | 50.71 (13.31) | 46.67 | 48.92 (5.83) | 42.86 | 48.74 (6.11) |

*AoA* = age of acquisition; *** = No standard deviations for patients since proportion of verbs represented.

**Table 2** Individual scores for every psycholinguistic variable for the non-pCMS-group (n = 7) and their controls for the conversational data.

| ***Level of language processing*** | ***Psycholinguistic variable*** | ***P17 (M(SD))*** | ***controls (M(SD))*** | ***P25 (M(SD))*** | ***controls (M(SD))*** | ***P24 (M(SD))*** | ***controls (M(SD))*** | ***P20 (M(SD))*** | ***controls (M(SD))*** |
| --- | --- | --- | --- | --- | --- | --- | --- | --- | --- |
| *Semantic* | *Imageability nouns* | 5.55 (1.28) | 6.17 (0.90) | 6.47 (0.28) | 5.92 (0.99) | 6.23 (0.60) | 5.84 (1.01) | 6.06 (0.95) | 5.69 (1.29) |
|  | *Imageability verbs* | 5.21 (1.24) | 4.43 (1.41) | 4.46 (1.70) | 4.87 (1.52) | 4.54 (1.45) | 4.44 (1.42) | 4.50 (1.22) | 4.34 (1.37) |
|  | *Concreteness nouns* | 3.50 (1.19) | 4.07 (0.96) | 4.13 (0.86) | 3.93 (0.93) | 3.39 (1.05) | 3.77 (1.01) | 3.94 (1.04) | 3.55 (1.12) |
|  | *Concreteness verbs* | 3.37 (1.00) | 2.57 (0.99) | 2.72 (1.22) | 2.88 (1.04) | 2.52 (1.03) | 2.74 (0.99) | 2.50 (0.79) | 2.69 (0.93) |
|  | *Verb instrumentality** | 28.57 | 14.05 (11.36) | 28.57 | 27.82 (16.88) | 12.50 | 13.74 (12.17) | 14.29 | 11.84 (8.13) |
| *Lexical* | *AoA nouns* | 4.43 (1.23) | 4.50 (1.13) | 4.80 (1.83) | 5.58 (1.09) | 4.92 (0.97) | 5.53 (1.28) | 5.81 (1.99) | 6.10 (1.83) |
|  | *AoA verbs* | 5.59 (1.30) | 5.00 (1.01) | 5.44 (1.43) | 5.31 (1.17) | 5.04 (0.68) | 5.60 (1.38) | 5.31 (1.52) | 5.69 (1.52) |
|  | *Word frequency nouns* | 170.14 (195.50) | 202.07 (229.08) | 267.41 (280.47) | 165.54 (233.16) | 134.95 (158.01) | 144.40 (191.89) | 157.23 (160.68) | 115.27 (166.60) |
|  | *Word frequency verbs* | 1322.29 (2941.98) | 1760.06 (2238.14) | 1659.72 (2854.96) | 1452.26 (2273.89) | 1361.81 (2074.25) | 1436.50 (2296.29) | 2430.61 (2833.89) | 1332.85 (2074.21) |
|  | *Phonological neighbourhood nouns* | 3.00 (4.00) | 7.40 (6.62) | 12.67 (7.04) | 8.20 (8.47) | 5.42 (5.90) | 7.38 (7.33) | 13.90 (11.10) | 7.32 (7.32) |
|  | *Phonological neighbourhood verbs* | 10.43 (6.80) | 11.14 (6.56) | 13.67 (7.15) | 10.37 (6.83) | 11.50 (6.76) | 11.35 (6.23) | 10.57 (8.48) | 9.58 (7.21) |
| *Phonological* | *Word length nouns* | 4.67 (1.03) | 4.48 (1.64) | 4.60 (2.41) | 5.35 (2.26) | 4.83 (1.99) | 5.02 (2.02) | 4.82 (2.18) | 5.06 (2.15) |
|  | *Word length verbs* | 5.29 (1.11) | 5.01 (1.22) | 5.29 (1.25) | 5.29 (1.63) | 5.31 (1.89) | 5.25 (1.55) | 4.86 (1.86) | 5.82 (1.87) |
| *Morphosyntactic* | *Verb regularity** | 42.86 | 25.27 (9.61) | 28.57 | 33.32 (24.59) | 12.50 | 32.90 (9.46) | 28.57 | 31.01 (18.13) |
|  | *Verb transitivity** | 57.14 | 56.32 (10.59) | 28.57 | 62.34 (7.02) | 50.00 | 54.64 (14.13) | 71.43 | 62.04 (11.09) |

*AoA* = age of acquisition; *** = No standard deviations for patients since proportion of verbs represented.

**Table 2 (continued)**

| ***Level of language processing*** | | ***Psycholinguistic variable*** | | ***P23 (M(SD))*** | | ***controls (M(SD))*** | | ***P26 (M(SD))*** | | ***controls (M(SD))*** | | ***P22 (M(SD))*** | | ***controls (M(SD))*** |  |
| --- | --- | --- | --- | --- | --- | --- | --- | --- | --- | --- | --- | --- | --- | --- | --- |
| *Semantic* | | *Imageability nouns* | | 4.54 (1.54) | | 5.23 (0.97) | | 5.67 (1.21) | | 5.20 (0.96) | | 4.12 (2.20) | | 4.75 (1.32) |  |
|  | | *Imageability verbs* | | 5.53 (1.28) | | 4.37 (1.30) | | 4.63 (1.59) | | 4.54 (1.29) | | 4.89 (1.08) | | 4.64 (1.37) |  |
|  | | *Concreteness nouns* | | 2.72 (1.25) | | 3.20 (0.97) | | 3.28 (0.83) | | 3.23 (0.96) | | 3.01 (0.93) | | 2.87 (1.07) |  |
|  | | *Concreteness verbs* | | 3.29 (0.86) | | 2.51 (0.86) | | 2.76 (1.17) | | 2.69 (0.95) | | 2.81 (0.96) | | 2.70 (0.93) |  |
|  | | *Verb instrumentality** | | 37.50 | | 8.89 (14.49) | | 42.86 | | 7.38 (12.38) | | 7.14 | | 8.86 (8.18) |  |
| *Lexical* | | *AoA nouns* | | 5.50 (1.28) | | 6.17 (1.26) | | 4.63 (0.81) | | 6.22 (1.31) | | 7.28 (2.56) | | 6.86 (2.58) |  |
|  | | *AoA verbs* | | 5.89 (2.48) | | 5.70 (1.29) | | 4.86 (0.70) | | 5.55 (1.31) | | 7.14 (2.56) | | 5.71 (1.35) |  |
|  | | *Word frequency nouns* | | 204.53 (252.07) | | 118.53 (200.44) | | 265.12 (278.68) | | 103.13 (176.22) | | 159.16 (217.54) | | 190.53 (242.66) |  |
|  | | *Word frequency verbs* | | 1248.17 (2739.40) | | 1761.93 (2686.55) | | 2112.24 (2929.50) | | 1648.66 (2472.72) | | 1236.64 (2161.51) | | 1147.01 (1912.86) |  |
|  | | *Phonological neighbourhood nouns* | | 8.38 (6.86) | | 7.39 (7.96) | | 9.75 (6.47) | | 7.81 (7.61) | | 4.92 (4.99) | | 7.78 (7.80) |  |
|  | | *Phonological neighbourhood verbs* | | 10.43 (7.37) | | 11.24 (7.99) | | 10.43 (8.10) | | 11.67 (7.54) | | 8.08 (7.52) | | 9.96 (7.03) |  |
| *Phonological* | | *Word length nouns* | | 4.50 (2.07) | | 6.10 (3.10) | | 4.38 (2.14) | | 6.19 (3.13) | | 5.43 (2.14) | | 5.63 (2.88) |  |
|  | | *Word length verbs* | | 6.13 (2.36) | | 5.35 (1.81) | | 5.00 (1.63) | | 5.38 (1.93) | | 6.07 (2.67) | | 5.75 (1.78) |  |
| *Morphosyntactic* | | *Verb regularity** | | 37.50 | | 29.05 (4.18) | | 28.57 | | 29.29 (7.74) | | 21.43 | | 26.57 (4.13) |  |
|  | *Verb transitivity** | | 37.50 | | 57.82 (15.42) | | 85.71 | | 52.02 (8.76) | | 42.86 | | 51.60 (9.22) | | |

*AoA* = age of acquisition; *** = No standard deviations for patients since proportion of verbs represented.

**Table 3** Individual scores for every psycholinguistic variable for the pCMS-group (n = 4) and their controls for the picture data.

| ***Level of language processing*** | ***Psycholinguistic variable*** | ***P6 (M(SD))*** | | ***controls (M(SD))*** | | ***P8 (M(SD))*** | | | ***controls (M(SD))*** | | ***P7 (M(SD))*** | ***controls (M(SD))*** | | ***P16 (M(SD))*** | | ***controls (M(SD))*** | |  |
| --- | --- | --- | --- | --- | --- | --- | --- | --- | --- | --- | --- | --- | --- | --- | --- | --- | --- | --- |
| *Semantic* | *Imageability nouns* | 6.29 (0.69) | | 6.30 (0.77) | | 6.21 (0.82) | | | 6.37 (0.69) | | 6.03 (1.09) | 5.94 (1.15) | | 6.41 (0.27) | | 5.94 (1.15) | |  |
|  | *Imageability verbs* | 4.77 (1.45) | | 5.07 (1.32) | | 4.90 (1.21) | | | 4.90 (1.34) | | 4.74 (1.48) | 5.01 (1.30) | | 5.03 (1.47) | | 5.01 (1.30) | |  |
|  | *Concreteness nouns* | 4.10 (0.97) | | 4.33 (0.70) | | 4.08 (0.86) | | | 4.33 (0.72) | | 4.07 (0.90) | 4.02 (0.88) | | 4.22 (0.64) | | 4.02 (0.88) | |  |
|  | *Concreteness verbs* | 2.86 (1.07) | | 3.16 (0.95) | | 2.98 (0.97) | | | 3.04 (0.99) | | 2.86 (0.97) | 3.12 (0.90) | | 3.17 (1.05) | | 3.12 (0.90) | |  |
|  | *Verb instrumentality** | 5.00 | | 13.63 (13.15) | | 0.00 | | | 16.12 (4.27) | | 6.90 | 12.14 (5.16) | | 21.43 | | 12.14 (5.16) | |  |
| *Lexical* | *AoA nouns* | 4.63 (1.17) | | 5.20 (1.31) | | 4.81 (1.36) | | | 5.32 (1.26) | | 5.85 (1.23) | 6.04 (1.67) | | 5.70 (1.48) | | 6.04 (1.67) | |  |
|  | *AoA verbs* | 5.15 (1.02) | | 5.62 (1.22) | | 5.01 (0.81) | | | 5.69 (1.14) | | 6.06 (1.88) | 6.08 (1.62) | | 5.87 (1.26) | | 6.08 (1.62) | |  |
|  | *Word frequency nouns* | 203.52 (244.94) | | 134.36 (172.72) | | 172.20 (189.27) | | | 155.08 (331.54) | | 111.16 (214.27) | 117.07 (223.24) | | 76.89 (175.48) | | 117.07 (223.24) | |  |
|  | *Word frequency verbs* | 1175.22 (1857.25) | | 1077.26 (1992.68) | | 1090.03 (1684.31) | | | 1062.68 (2044.83) | | 797.48 (1602.23) | 795.96 (1701.66) | | 948.39 (2104.41) | | 795.96 (1701.66) | |  |
|  | *Phonological neighbourhood nouns* | 8.35 (9.13) | | 7.40 (7.53) | | 8.43 (9.82) | | | 8.03 (8.23) | | 8.31 (8.38) | 6.52 (7.55) | | 6.06 (6.81) | | 6.52 (7.55) | |  |
|  | *Phonological neighbourhood verbs* | 12.16 (6.76) | | 11.47 (7.56) | | 10.91 (6.49) | | | 10.16 (7.81) | | 9.71 (6.39) | 9.26 (7.30) | | 10.07 (8.25) | | 9.26 (7.30) | |  |
| *Phonological* | *Word length nouns* | 4.95 (1.73) | | 5.25 (2.19) | | 4.93 (1.82) | | | 5.11 (1.93) | | 5.87 (2.90) | 5.74 (2.35) | | 5.58 (1.95) | | 5.74 (2.35) | |  |
|  | *Word length verbs* | 5.05 (1.19) | | 5.43 (1.83) | | 5.21 (1.10) | | | 5.70 (1.89) | | 5.76 (1.60) | 5.78 (1.83) | | 5.93 (2.27) | | 5.78 (1.83) | |  |
| *Morphosyntactic* | *Verb regularity** | 20.00 | | 32.62 (12.14) | | 20.83 | | | 37.68 (7.73) | | 34.48 | 35.89 (8.57) | | 42.86 | | 35.89 (8.57) | |  |
|  | *Verb transitivity** | | 50.00 | | 60.18 (19.20) | | 58.33 | 46.26 (9.67) | | 44.83 | | | 47.64 (5.55) | | 50.00 | | 47.64 (5.55) | |

*AoA* = age of acquisition; *** = No standard deviations for patients since proportion of verbs represented.

**Table 4** Individual scores for every psycholinguistic variable for the non-pCMS-group (n = 7) and their controls for the picture data.

| ***Level of language processing*** | | ***Psycholinguistic variable*** | ***P17 (M(SD))*** | | | ***controls (M(SD))*** | ***P25 (M(SD))*** | | ***controls (M(SD))*** | | ***P24 (M(SD))*** | | ***controls (M(SD))*** | | ***P20 (M(SD))*** | | | ***controls (M(SD))*** | |
| --- | --- | --- | --- | --- | --- | --- | --- | --- | --- | --- | --- | --- | --- | --- | --- | --- | --- | --- | --- |
| *Semantic* | | *Imageability nouns* | 6.44 (0.25) | | | 6.42 (0.60) | 6.30 (0.74) | | 6.29 (0.69) | | 6.29 (0.82) | | 6.23 (0.80) | | 5.71 (1.36) | | | 6.25 (0.76) | |
|  | | *Imageability verbs* | 5.11 (1.51) | | | 4.70 (1.42) | 5.26 (1.55) | | 4.77 (1.45) | | 5.41 (1.29) | | 5.05 (1.29) | | 4.46 (0.90) | | | 4.83 (1.36) | |
|  | | *Concreteness nouns* | 4.71 (0.24) | | | 4.38 (0.63) | 4.33 (0.74) | | 4.10 (0.97) | | 4.29 (0.67) | | 4.25 (0.78) | | 3.71 (1.13) | | | 4.21 (0.76) | |
|  | | *Concreteness verbs* | 3.23 (1.08) | | | 2.91 (1.07) | 3.15 (1.26) | | 2.86 (1.07) | | 3.23 (0.92) | | 3.14 (0.98) | | 2.67 (0.93) | | | 2.97 (1.04) | |
|  | | *Verb instrumentality** | 0.00 | | | 2.58 (3.54) | 11.11 | | 5.00 | | 11.76 | | 14.49 (4.90) | | 0.00 | | | 6.57 (6.75) | |
| *Lexical* | | *AoA nouns* | 5.37 (0.81) | | | 4.88 (1.31) | 5.16 (1.31) | | 4.63 (1.17) | | 5.23 (1.14) | | 5.54 (1.37) | | 5.59 (1.10) | | | 5.63 (1.38) | |
|  | | *AoA verbs* | 4.86 (0.77) | | | 5.24 (1.08) | 4.99 (1.18) | | 5.15 (1.02) | | 5.52 (1.19) | | 5.76 (1.51) | | 5.47 (0.85) | | | 5.60 (1.33) | |
|  | | *Word frequency nouns* | 81.60 (126.32) | | | 201.20 (283.76) | 192.02 (261.42) | | 203.52 (244.94) | | 271.87 (552.43) | | 109.60 (175.31) | | 178.46 (252.90) | | | 92.48 (166.75) | |
|  | | *Word frequency verbs* | 1379.95 (1973.39) | | | 1395.99 (2279.11) | 1549.20 (2632.57) | | 1175.22 (1857.25) | | 803.45 (1895.87) | | 1089.67 (2013.86) | | 1473.13 (2358.77) | | | 1124.61 (2082.39) | |
|  | | *Phonological neighbourhood nouns* | 8.43 (9.31) | | | 7.66 (7.07) | 8.69 (7.24) | | 8.35 (9.13) | | 8.83 (7.77) | | 7.99 (8.32) | | 8.91 (9.51) | | | 7.75 (7.64) | |
|  | | *Phonological neighbourhood verbs* | 14.25 (5.30) | | | 12.09 (7.43) | 12.78 (7.29) | | 12.16 (6.76) | | 11.45 (7.63) | | 9.76 (7.59) | | 12.55 (6.85) | | | 10.59 (7.77) | |
| *Phonological* | | *Word length nouns* | 5.14 (1.96) | | | 4.82 (1.77) | 4.67 (1.14) | | 4.95 (1.73) | | 4.87 (2.16) | | 5.33 (2.07) | | 4.86 (1.49) | | | 5.59 (2.29) | |
|  | | *Word length verbs* | 4.50 (1.10) | | | 5.14 (1.57) | 5.11 (1.62) | | 5.05 (1.19) | | 5.53 (2.07) | | 5.77 (2.20) | | 4.82 (1.33) | | | 5.47 (1.97) | |
| *Morphosyntactic* | | *Verb regularity** | 12.50 | | | 17.21 (4.62) | 22.22 | | 20.00 | | 23.53 | | 29.47 (10.37) | | 36.36 | | | 25.55 (9.27) | |
|  | *Verb transitivity** | | | 50.00 | 51.25 (11.91) | | | 66.67 | | 50.00 | | 41.18 | | 53.99 (6.91) | | 63.64 | 53.44 (12.34) | |  |

*AoA* = age of acquisition; *** = No standard deviations for patients since proportion of verbs represented.

**Table 4 (continued)**

| ***Level of language processing*** | ***Psycholinguistic variable*** | ***P23 (M(SD))*** | ***controls (M(SD))*** | ***P26 (M(SD))*** | ***controls (M(SD))*** | ***P22 (M(SD))*** | ***controls (M(SD))*** |
| --- | --- | --- | --- | --- | --- | --- | --- |
| *Semantic* | *Imageability nouns* | 6.31 (0.64) | 6.25 (0.88) | 6.36 (0.48) | 6.25 (0.88) | 5.33 (2.24) | 6.05 (1.03) |
|  | *Imageability verbs* | 5.09 (1.30) | 5.11 (1.34) | 5.06 (1.29) | 5.11 (1.34) | 4.83 (1.42) | 4.92 (1.37) |
|  | *Concreteness nouns* | 4.26 (0.72) | 4.30 (0.79) | 4.30 (0.62) | 4.30 (0.79) | 3.94 (0.71) | 3.92 (1.01) |
|  | *Concreteness verbs* | 3.11 (1.02) | 3.16 (0.97) | 3.14 (0.97) | 3.16 (0.97) | 2.89 (1.00) | 3.08 (0.94) |
|  | *Verb instrumentality** | 17.64705882 | 10.98 (3.29) | 12.5 | 10.98 (3.29) | 11.76470588 | 14.78 (4.03) |
| *Lexical* | *AoA nouns* | 5.25 (1.09) | 5.68 (1.39) | 5.56 (1.28) | 5.68 (1.39) | 5.68 (1.27) | 5.68 (1.69) |
|  | *AoA verbs* | 6.09 (1.51) | 5.63 (1.35) | 5.43 (1.20) | 5.63 (1.35) | 5.40 (1.11) | 6.06 (1.54) |
|  | *Word frequency nouns* | 148.63 (297.74) | 100.32 (192.17) | 108.28 (264.10) | 100.32 (192.17) | 52.74 (99.57) | 152.04 (267.87) |
|  | *Word frequency verbs* | 896.15 (2018.00) | 881.82 (1833.60) | 1096.69 (2036.36) | 881.82 (1833.60) | 1202.41 (1991.47) | 839.72 (1799.04) |
|  | *Phonological neighbourhood nouns* | 7.87 (7.92) | 8.75 (9.14) | 8.14 (8.26) | 8.75 (9.14) | 4.91 (7.65) | 7.04 (7.38) |
|  | *Phonological neighbourhood verbs* | 8.59 (7.88) | 10.58 (7.25) | 10.00 (6.82) | 10.58 (7.25) | 10.82 (6.84) | 9.48 (7.29) |
| *Phonological* | *Word length nouns* | 5.13 (2.06) | 5.12 (2.20) | 5.02 (1.94) | 5.12 (2.20) | 5.83 (1.97) | 5.51 (2.29) |
|  | *Word length verbs* | 5.88 (2.03) | 5.69 (1.85) | 5.31 (1.08) | 5.69 (1.85) | 5.35 (2.09) | 5.84 (1.89) |
| *Morphosyntactic* | *Verb regularity** | 41.17647059 | 23.71 (7.02) | 25 | 23.71 (7.02) | 29.41176471 | 32.89 (2.51) |
|  | *Verb transitivity** | 52.94 | 41.96 (5.92) | 43.75 | 41.96 (5.92) | 47.06 | 47.39 (8.07) |

*AoA* = age of acquisition; *** = No standard deviations for patients since proportion of verbs represented.
